# The Use of Generative AI for Scientific Literature Searches for Systematic Reviews: ChatGPT and Microsoft Bing AI Performance Evaluation

**DOI:** 10.2196/51187

**Published:** 2024-05-14

**Authors:** Yong Nam Gwon, Jae Heon Kim, Hyun Soo Chung, Eun Jee Jung, Joey Chun, Serin Lee, Sung Ryul Shim

**Affiliations:** 1 Department of Urology, Soonchunhyang University College of Medicine, Soonchunhyang University Seoul Hospital, Seoul, Republic of Korea; 2 College of Medicine, Soonchunhyang University, Cheonan, Republic of Korea; 3 Cranbrook Kingswood Upper School, Bloomfield Hills, MI, United States; 4 Department of Biochemistry, Case Western Reserve University, Cleveland, OH, United States; 5 Department of Biomedical Informatics, Konyang University College of Medicine, Daejeon, Republic of Korea; 6 Konyang Medical Data Research Group-KYMERA, Konyang University Hospital, Daejeon, Republic of Korea

**Keywords:** artificial intelligence, search engine, systematic review, evidence-based medicine, ChatGPT, language model, education, tool, clinical decision support system, decision support, support, treatment

## Abstract

**Background:**

A large language model is a type of artificial intelligence (AI) model that opens up great possibilities for health care practice, research, and education, although scholars have emphasized the need to proactively address the issue of unvalidated and inaccurate information regarding its use. One of the best-known large language models is ChatGPT (OpenAI). It is believed to be of great help to medical research, as it facilitates more efficient data set analysis, code generation, and literature review, allowing researchers to focus on experimental design as well as drug discovery and development.

**Objective:**

This study aims to explore the potential of ChatGPT as a real-time literature search tool for systematic reviews and clinical decision support systems, to enhance their efficiency and accuracy in health care settings.

**Methods:**

The search results of a published systematic review by human experts on the treatment of Peyronie disease were selected as a benchmark, and the literature search formula of the study was applied to ChatGPT and Microsoft Bing AI as a comparison to human researchers. Peyronie disease typically presents with discomfort, curvature, or deformity of the penis in association with palpable plaques and erectile dysfunction. To evaluate the quality of individual studies derived from AI answers, we created a structured rating system based on bibliographic information related to the publications. We classified its answers into 4 grades if the title existed: A, B, C, and F. No grade was given for a fake title or no answer.

**Results:**

From ChatGPT, 7 (0.5%) out of 1287 identified studies were directly relevant, whereas Bing AI resulted in 19 (40%) relevant studies out of 48, compared to the human benchmark of 24 studies. In the qualitative evaluation, ChatGPT had 7 grade A, 18 grade B, 167 grade C, and 211 grade F studies, and Bing AI had 19 grade A and 28 grade C studies.

**Conclusions:**

This is the first study to compare AI and conventional human systematic review methods as a real-time literature collection tool for evidence-based medicine. The results suggest that the use of ChatGPT as a tool for real-time evidence generation is not yet accurate and feasible. Therefore, researchers should be cautious about using such AI. The limitations of this study using the generative pre-trained transformer model are that the search for research topics was not diverse and that it did not prevent the hallucination of generative AI. However, this study will serve as a standard for future studies by providing an index to verify the reliability and consistency of generative AI from a user’s point of view. If the reliability and consistency of AI literature search services are verified, then the use of these technologies will help medical research greatly.

## Introduction

The global artificial intelligence (AI) health care market size was estimated to be at US $15.1 billion in 2022 and is expected to surpass approximately US $187.95 billion by 2030, growing at an annualized rate of 37% during the forecast period from 2022 to 2030 [1]. In particular, innovative applications of medical AI are expected to increase in response to medical demand, which will explode in 2030 [2,3].

A large language model (LLM) is a type of AI model that opens up great possibilities for health care practice, research, and education, although scholars have emphasized the need to proactively address the issue of unvalidated and inaccurate information regarding its use [4,5]. One of the best-known LLMs is ChatGPT (OpenAI). It was launched in November 2022. Similar to other LLMs, ChatGPT is trained on huge text data sets in numerous languages, allowing it to respond to text input with humanlike responses [4]. Developed by the San Francisco–based AI research laboratory OpenAI, ChatGPT is based on a generative pre-trained transformer (GPT) architecture. It is considered an advanced form of a chatbot, an umbrella term for a program that uses a text-based interface to understand and generate responses. The key difference between a chatbot and ChatGPT is that a chatbot is usually programmed with a limited number of responses, whereas ChatGPT can produce personalized responses according to the conversation [4,6].

Sallam’s [5] systematic review (SR) sought to identify the benefits and current concerns regarding ChatGPT. That review advises that health care research could benefit from ChatGPT, since it could be used to facilitate more efficient data set analysis, code generation, and literature reviews, thus allowing researchers to concentrate on experimental design as well as drug discovery and development. The author also suggests that ChatGPT could be used to improve research equity and versatility in addition to its ability to improve scientific writing. Health care practice could also benefit from ChatGPT in multiple ways, including enabling improved health literacy and delivery of more personalized medical care, improved documentation, workflow streamlining, and cost savings. Health care education could also use ChatGPT to provide more personalized learning with a particular focus on problem-solving and critical thinking skills [5]. However, the same review also lays out the current concerns, including copyright issues, incorrect citations, and increased risk of plagiarism, as well as inaccurate content, risk of excessive information leading to an infodemic on a particular topic, and cybersecurity issues [5].

A key question regarding the use of ChatGPT is if it can use evidence to identify premedical content. Evidence-based medicine (EBM) provides the highest level of evidence in medical treatment by integrating clinician experience, patient value, and best-available scientific information to guide decision-making on clinical management [7]. The principle of EBM means that the most appropriate treatment plan for patients should be devised based on the latest empirical research evidence. However, the scientific information identified by ChatGPT is not yet validated in terms of safety or accuracy according to Sallam [5], who further suggests that neither doctors nor patients should rely on it at this stage. In contrast, another study by Zhou et al [8] found that answers provided by ChatGPT were generally based on the latest verified scientific evidence, that is, the advice given followed high-quality treatment protocols and adhered to guidelines from experts.

In medicine, a clinical decision support system (CDSS) uses real-time evidence to support clinical decision-making. This is a fundamental tool in EBM, which uses SRs based on a systematic, scientific search of a particular subject. If ChatGPT becomes a CDSS, it is fundamental to determine whether it is capable of performing a systematic search based on real-time generation of evidence in the medical field. Therefore, this study will be the first to determine whether ChatGPT can search papers for an SR. In particular, this study aims to present a standard for medical research using generative AI search technology in the future by providing indicators for the reliability and consistency of generative AI searches from a user’s perspective.

## Methods

### Ethical Considerations

As per 45 CFR §46.102(f), the activities performed herein were considered exempt from institutional review board approval due to the data being publicly available. Informed consent was not obtained, since this study used previously published deidentified information that was available to the general public. This study used publicly available data from PubMed, Embase, and Cochrane Library and did not include human participant research.

### Setting the Benchmark

To determine whether ChatGPT, currently the most representative LLM, is capable of systematic searches, we set an SR that was performed by human experts as a benchmark and checked how many studies were finally included in the benchmark were presented by ChatGPT. We chose Lee et al [9] as the benchmark for the following reasons. First, Lee et al [9] performed an SR and meta-analysis about the medical treatment for Peyronie disease (PD) with human experts. PD typically presents with discomfort, curvature, or deformity of the penis in association with palpable plaques and erectile dysfunction [10]. Second, it was easy to compare the results of ChatGPT and the benchmark, because we had full information about the interim process and results of the study. Third, a sufficient amount of studies has been published about the medical treatment for PD, but there is still no consensus answer. So, we expected to assess the sole ability of ChatGPT as a systematic search tool with sufficient data while avoiding any possible pretrained bias. Lastly, with the topic of Lee et al [9], we could build questions that start broad and become more specific and add some conditions that could test ChatGPT’s comprehension about scientific research. For example, questions could not only be built broadly by asking about “medical treatment for Peyronie’s disease” but also specifically by asking about “oral therapy for Peyronie’s disease” or “colchicine for Peyronie’s disease.” Because Lee et al [9] only contained randomized controlled trials (RCTs), we could add a condition to the questions to restrict the study type to RCTs, which could be useful to assess the comprehension of ChatGPT.

### Systematic Search Formula of Benchmark

**Figure 1.**
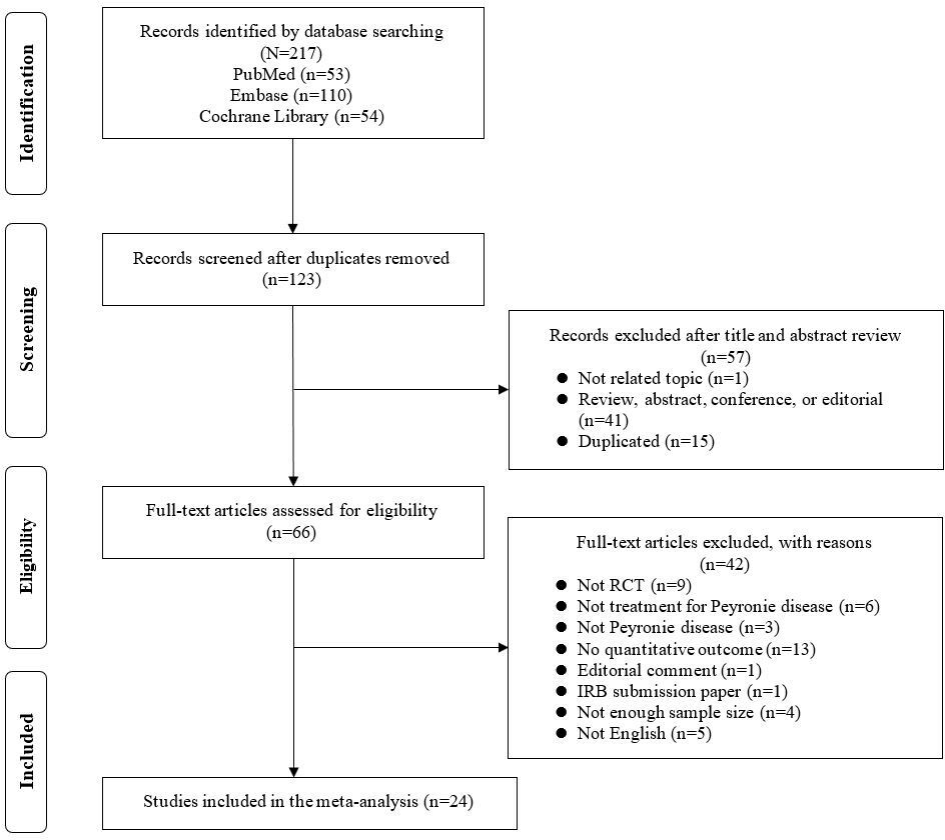
PRISMA (Preferred Reporting Items for Systematic Reviews and Meta-Analyses) flowchart for Lee et al [9]. RCT: randomized controlled trial.

Lee et al [9] used the following search query in PubMed and Cochrane Library: *(“penile induration”[MeSH Terms] OR “Peyronie’s disease”[Title/Abstract]) AND “male”[MeSH Terms] AND “randomized controlled trial”[Publication Type]*, and the following query in Embase: *(‘Peyronie disease’/exp OR ’Peyronie’s diseas’:ab,ti) AND ’male’/exp AND ’randomized controlled trial’/de*. After the systematic search, a total of 217 records were identified. Studies were excluded for the following reasons: not RCTs, not perfectly fit to the topic, not enough sample size or outcome, and not written in English. Finally, 24 RCTs were included in the SR, with only 1 RCT published in 2022 (Figure 1) [9]. The characteristics of all studies included in Lee et al [9] are summarized in Section S1 in Multimedia Appendix 1.

### Methodology of Systematic Search for ChatGPT

**Figure 2.**
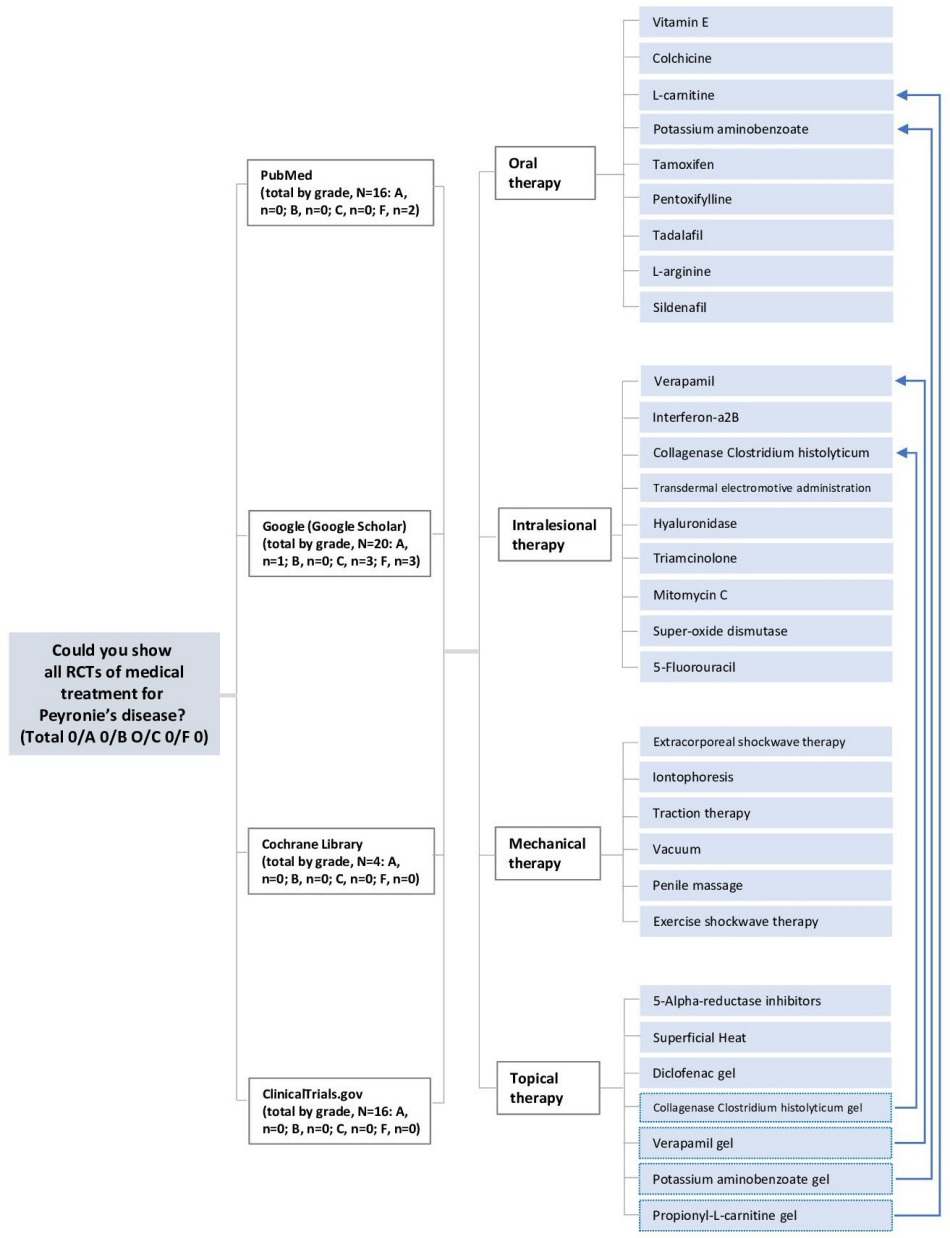
Logical flow and results focusing on database for ChatGPT. RCT: randomized controlled trial.

Based on the search formula used in Lee et al [9], a simple mandatory prompt in the form of a question was created, starting with comprehensive questions and gradually asking more specific questions (Textbox 1). For example, questions could be built as “Could you show RCTs of colchicine for Peyronie’s disease in PubMed?” with the treatment and database changed under the same format. In addition to mandatory questions, we added questions about treatment additionally provided by ChatGPT during the conversation. Considering the possibility that ChatGPT might respond differently depending on the interaction, we arranged questions into 2 logical flows, focusing on database and treatment, respectively (Figure 2 and Figure S1 in Multimedia Appendix 1). We asked about search results from 4 databases: PubMed [11], Google (Google Scholar) [12], Cochrane Library [13], and ClinicalTrials.gov [14]. PubMed is a leading biomedical database offering access to peer-reviewed articles. Google Scholar provides a wide-ranging index of scholarly literature, including medical studies. Cochrane Library specializes in high-quality evidence through SRs and clinical trials. ClinicalTrials.gov, managed by the National Library of Medicine, serves as a comprehensive repository for clinical study information globally. These databases collectively serve researchers by providing access to diverse and credible sources, facilitating literature reviews and evidence synthesis, and informing EBM in the medical field. They play crucial roles in advancing medical knowledge, supporting informed decision-making, and ultimately improving patient care outcomes [11-14]. These 4 databases were easy to access and contained most of the accessible studies. Each question was repeated at least twice. We extracted the answers and evaluated the quality of information based on the title, author, journal, and publication year (Sections S2-S5 Multimedia Appendix 1).

Textbox 1. **Mandatory question prompts.**

**Basic format of questions**

- “Could you show RCTs of (A) for Peyronie’s disease in (B)?”

**(A) Treatment category and specific treatment**

- **Oral therapy**
  - Vitamin E, colchicine, L-carnitine, potassium aminobenzoate, tamoxifen, pentoxifylline, tadalafil, L-arginine, and sildenafil
- **Intralesional therapy**
  - Verapamil, interferon-a2B, collagenase *Clostridium histolyticum*, transdermal electromotive administration, hyaluronidase, triamcinolone, mitomycin C, super-oxide dismutase, and 5-fluorouracil
- **Mechanical therapy**
  - Extracorporeal shockwave therapy, iontophoresis, traction therapy, vacuum, penile massage, and exercise shockwave therapy
- **Topical therapy**
  - 5-Alpha-reductase inhibitors, superficial heat, diclofenac gel, collagenase *Clostridium histolyticum* gel, verapamil gel, potassium aminobenzoate gel, and propionyl-L-carnitine gel

**(B) Database**

- PubMed
- Google (Google Scholar)
- Cochrane Library
- ClinicalTrials.gov

We used the GPT-3.5 version of ChatGPT, which was pretrained with data before 2021, for the systematic search and evaluated how many RCTs that were included in Lee et al [9] were present in the search results from ChatGPT. To assess the reliability of ChatGPT’s answers, we also evaluated whether the studies presented actually existed. ChatGPT’s response style and the amount of information might vary from answer to answer. Thus, we evaluated the accuracy of the responses by prioritizing a match by (1) title; (2) author, journal, and publication year; and (3) other items.

To obtain higher-quality responses, it is important to structure the prompts using refined language that is well understood by the LLM [15-17]. In this study, we performed the following fine-tuning to clearly convey the most important content or information. We first defined roles and provided context and input data before asking complete questions to get responses, and we used specific and varied examples to help the model narrow its focus and produce more accurate results [18,19]. During the prompt engineering, the treatment category, specific treatment, and target databases were structured in order, and the order was changed in the detailed elements to induce consistent answers. Details of this are presented in Multimedia Appendix 1.

### Quality Assessment of Answers

To evaluate the quality of individual studies derived from AI answers, we created a structured rating system based on bibliographic information related to the publications (Table 1). We classified its answers into 4 grades if the title existed: A, B, C, and F. No grade was given for a fake title or no answer.

**Table 1.** Grade table based on bibliographic information.

Grade	Title actually exists	PICOS^a^	Essential information				Accessory information				Definition of grade
			Title	Author	Journal	Publication year	Issue number	Page number	DOI	PMID	
A	Yes	✓^b^	✓	✓	✓	✓	✓	✓	✓	✓	All bibliographic information matched
B	Yes	✓	✓	✓	✓	✓	Any X^c^	Any X	Any X	Any X	PICOS and essential information matched, but not accessory information
C	Yes	X^d^	✓	✓	✓	✓	N/A^e^	N/A	N/A	N/A	Essential information matched, but not PICOS
F	Yes	N/A	✓	Any X	Any X	Any X	N/A	N/A	N/A	N/A	Title matched, but not other essential information

a PICOS: population, intervention, comparison, outcome, and study design (research questions).

b Matched.

c Any mismatch in essential information or accessory information.

d Mismatch.

e N/A: not assessed.

A grade of “A” was given to an answer that was appropriate for the question and perfectly consistent with the actual study. For example, for the question “Could you show all RCTs of medical treatment for Peyronie’s disease in Google?” ChatGPT answered “Gelbard MK, James K, Riach P, Dorey F. Collagenase versus placebo in the treatment of Peyronie’s disease: a double-blind study. J Urol. 1993; 149(1):56-58.” This answer was perfectly consistent with actual study because the authors, title, journal, publication year, issue number, and even page number matched, and it was appropriate because it was an RCT, was about medical treatment for PD, and could be found in Google.

A grade of “B” was given to an answer that was appropriate for the question and the title, author, journal, and publication year matched with the actual study. For example, for the question “Could you show RCTs of Intralesional therapy for Peyronie’s disease in Cochrane Library?” ChatGPT answered “Gelbard MK, James K, Riach P, Dorey F. Collagenase versus placebo in the treatment of Peyronie’s disease: a double-blind study. J Urol. 1993 Feb; 149(2):56-8. doi: 10.1016/s0022-5347(17)36148-1. PMID: 8426418.” This answer was appropriate because it was an RCT, was about intralesional therapy for PD, and could be found in Cochrane Library. However, this answer was consistent with the title, authors, journal, and publication year of actual study but inconsistent with the accessory information for publication month, issue number, page number, DOI, and PMID. So, it was able to specify the presented study with essential information, but the accessory information was not consistent with the actual study.

A grade of “C” was given to an answer that was inappropriate for the question but the title, author, journal, and publication year matched with the actual study. For example, for the question “Could you show RCTs of Pentoxifylline for Peyronie’s disease in PubMed?” ChatGPT answered “Gelbard MK, James K, Riach P, Dorey F. Collagenase versus placebo in the treatment of Peyronie’s disease: a double-blind study. J Urol. 1993; 149(1):56-8. doi: 10.1016/s0022-5347(17)36091-7. PMID: 8417228.” This answer was consistent with the title, authors, journal, and publication year of the actual study, but it was inappropriate because it was not about the use of pentoxifylline for PD.

A grade of “F” was given to an answer that was inconsistent with the author, journal, or publication year of the actual study that matched the title, making it impossible to be specified. Thus, it was considered as a fake study that was generated. For example, for the question “Could you show RCTs of collagenase *Clostridium histolyticum* for Peyronie’s disease in PubMed?” ChatGPT answered “Gelbard MK, James K, Riach P, Dorey FJ, & Collagenase Study Group. (2012). Collagenase versus placebo in the treatment of Peyronie’s disease: a double-blind study. The Journal of urology, 187(3), 948-953.” This answer was consistent with the title of the actual study but inconsistent with the authors, publication year, and so on.

### Searching Strategy for Bing AI

To compare with ChatGPT, we performed the same process with Bing AI [20], also known as “New Bing,” an AI chatbot developed by Microsoft and released in 2023. Since Bing AI functions based on the huge AI model “Prometheus” that includes OpenAI’s GPT-4 with web searching capabilities, it is expected to give more accurate answers than the GPT-3.5 version of ChatGPT. We performed the conversation with the “Precise” tone. Because Bing AI limited the number of questions per session to 20, we did not arrange questions into 2 logical flows (Section S6 in Multimedia Appendix 1). We compared the number of studies included in the benchmark [9] and provided by Bing AI. We also evaluated the reliability of answers with the same method described above or using links of websites presented by Bing AI (Figure S2 and Section S7 in Multimedia Appendix 1).

## Results

### Systematic Search Results via ChatGPT

A total of 639 questions were entered into ChatGPT, and 1287 studies were obtained (Table 2). The systematic search via ChatGPT was performed from April 17 to May 6, 2023. At the beginning of the conversation, we gave ChatGPT the role of a researcher conducting a systematic search who intended to perform a meta-analysis for more appropriate answers. At first, we tried to build question format by using the word “find,” such as “Could you find RCTs of medical treatment for Peyronie’s disease?” However, ChatGPT did not present studies and only suggested how to find RCTs in a database, such as PubMed. Therefore, we changed the word “find” to “show,” and ChatGPT presented lists of RCTs. For comprehensive questions, ChatGPT did not give an answer, saying that it did not have the capability to show a list of RCTs as an AI language model. However, when questions were gradually specified, it created answers (Sections S2 and S4 in Multimedia Appendix 1).

**Table 2.** Quality assessment of answers from ChatGPT and Bing AI^a^.

Searcher, setting, and question level			Grade, n				Studies, n
			A	B	C	F	
**ChatGPT**							
	**Database setting**						
		Comprehensive question	1	0	3	5	56
		Category-specific question	1	1	8	18	124
		Treatment-specific question	4	7	67	87	545
		Total	6	8	78	110	725
	**Treatment setting**						
		Comprehensive question	0	0	0	1	27
		Category-specific question	0	0	4	8	61
		Treatment-specific question	1	10	85	92	474
		Total	1	10	89	101	562
	Total		7	18	167	211	1287
**Bing AI**							
	Comprehensive question		0	0	1	0	1
	Category-specific question		0	0	7	0	7
	Treatment-specific question		19	0	20	0	40
	Total		19	0	28	0	48
Human^b^			24	0	0	0	24

a AI: artificial intelligence.

b From Lee et al [9].

Of the 1287 studies provided by ChatGPT, only 7 (0.5%) studies were perfectly eligible and 18 (1.4%) studies could be considered suitable under the assumption that they were real studies if only the title, author, journal, and publication year matched (Table 2). Among these, only 1 study was perfectly consistent with studies finally included in Lee et al [9], and 4 studies were matched under the assumption (Sections S1, S3, and S5 in Multimedia Appendix 1).

Specifically, systematic search via ChatGPT was performed in 2 logical flow schemes, database setting and treatment setting (Figure 2 and Figure S1 in Multimedia Appendix 1). With the logical flow by database setting, among the 725 obtained studies, 6 (0.8%) and 8 (1.1%) studies were classified as grade A and grade B, respectively (Table 1). Of these, 1 grade A study and 1 grade B study were included in Lee et al [5]. With the logical flow by treatment setting, among the 562 obtained studies, 1 (0.2%) study was classified as grade A and 10 (1.8%) studies were classified as grade B. Of these, 3 grade B studies were included in the benchmark [9] (Table 2).

It was common for answers to be changed. There were many cases where answers contradicted themselves. In addition, there were cases where the answer was “no capability” or “no RCT found” at first, but when another question was asked and the previous question was asked again, an answer was given. ChatGPT showed a tendency to create articles by rotating some format and words. Titles presented were so plausible that it was almost impossible to identify fake articles until an actual search was conducted. The presented authors were also real people. Titles often contained highly specific numbers, devices, or brand names that were real. There were some cases where it was possible to infer which articles ChatGPT mimicked in the fake answers (Sections S3 and S5 in Multimedia Appendix 1). Considering these characteristics, when generating sentences, ChatGPT seemed to list words with a high probability of appearing among pretrained data rather than presenting accurate facts or understanding questions.

In conclusion, of the 1287 studies presented by ChatGPT, only 1 (0.08%) RCT matched the 24 RCTs of the benchmark [9].

### Systematic Search Results via Bing AI

For Bing AI, a total of 223 questions were asked and 48 studies were presented. Among the 48 obtained studies, 19 (40%) studies were classified as grade A. There were no grade B studies (Table 2). Because Bing AI always gave references with links to the websites, all studies presented by Bing AI existed. However, it also provided wrong answers about the study type, especially as it listed reviews as RCTs. Of the 28 studies with grade C, 27 (96%) were not RCTs and 1 (4%) was about a different treatment. Only 1 study had no grade because of a fake title; it presented a study registered in PubMed while pretending that it was the result of a search in ClinicalTrials.gov. However, the study was not in ClinicalTrials.gov (Section S7 in Multimedia Appendix 1).

Bing AI had more accurate answers than ChatGPT since it provides actual website references. However, it also showed a tendency to give more answers to more specific questions, similar to ChatGPT. For example, with a comprehensive question, Bing AI said “I am not able to access or search specific databases.” However, with more specific questions, it found studies or answered “I couldn’t find any RCTs’ without mention about accessibility.” In most cases, Bing AI either failed to find studies or listed too few studies to be used as a systematic searching tool.

In conclusion, of the 48 studies presented by Bing AI, 2 (4%) RCTs matched the 24 RCTs of the benchmark [9].

## Discussion

### Principal Findings

This paper’s researchers sought to determine whether ChatGPT could conduct a real-time systematic search for EBM. For the first time, researchers compared the performance of ChatGPT with classic systematic searching as well as the Microsoft Bing AI search engine. Although Zhou et al [8] suggested that ChatGPT answered qualitative questions based on recent evidence, this study found that ChatGPT’s results were not based on a systematic search (which is the basis for an SR), meaning that they could not be used for real-time CDSS in their current state.

With recent controversy regarding the risks and benefits of advanced AI technologies [21-24], ChatGPT has received mixed responses from the scientific community and academia. Although many scholars agree that ChatGPT can increase the efficiency and accuracy of the output in writing and conversational tasks [25], others suggest that the data sets used in ChatGPT’s training might lead to possible bias, which not only limits its capabilities but also leads to the phenomenon of hallucination—apparently scientifically plausible yet factually inaccurate information [24]. Caution around the use of LLMs should also bear in mind security concerns, including the potential of cyberattacks that deliberately spread misinformation [25].

When applying the plug-in method in this study, especially when using PubMed Research [26], the process worked smoothly and there was not a single case of hallucination of fake research (by providing information along with a link), regardless of the designation of a specific database engine. Among the responses, 21 RCTs were included in the final SR, and out of a total of 24, all RCTs except 3 were provided. This is a very encouraging result. However, there is no plug-in that allows access to other databases yet, and if the conversation is long, the response speed is very slow. Furthermore, although it is a paid service, it only provides a total of 100 papers, so if more than 100 RCTs are searched, the user must manually search all papers. Ultimately, it is not intended for conducting an efficient and systematic search, as additional time and effort are required. If a more efficient plug-in is developed, this could play a promising part in systematic searches.

Although Sallam’s [5] SR suggests that academic and scientific writing as well as health care practice, research, and education could benefit from the use of ChatGPT, this study found that ChatGPT could not search scientific articles properly, with a 0.08% (1/1287) of probability of the desired paper being presented. In the case of Bing AI using GPT-4, this study showed that Bing AI could search scientific articles with a much higher accuracy than ChatGPT. However, the probability was only 4% (2/48). It was still an insufficient probability for performing systematic research. Moreover, fake answers generated by ChatGPT, known as hallucinations, caused researchers to spend extra time and effort by checking the accuracy of the answers. A typical problem with generative AI is that it creates hallucinations. However, this is difficult to completely remove due to the principle of generative AI. Therefore, if it cannot be prevented from the pretraining of the model, efforts to increase reliability and consistency in the use of generative AI in medical care by checking the accuracy from the user’s point of view are required, as shown in this study. Unlike ChatGPT, Bing AI did not generate fake studies. However, the total number of studies presented was too small. Very few studies have focused on the scientific searching accuracy of ChatGPT. Although this paper found many articles about the use of ChatGPT in the medical field, the majority concerned the role of ChatGPT as an author. Although the latter might accelerate writing efficiency, it also confirms the previously mentioned issues of transparency and plagiarism.

Wang et al [27] have recently investigated whether ChatGPT could be used to generate effective Boolean queries for an SR literature search. The authors suggest that ChatGPT should be considered a “valuable tool” for researchers conducting SRs, especially for time-constrained rapid reviews where trading off higher precision for lower recall is generally acceptable. They cite its ability to follow complex instructions and generate high-precision queries. Nonetheless, it should be noted that building a Boolean query is not a complex process. However, selecting the most appropriate articles for an SR is critical, which might be a more useful subject to examine in relation to the use of ChatGPT. Moreover, although Aydın and Karaarslan [28] have indicated that ChatGPT shows promise in generating a literature review, the iThenticate plagiarism tool found significant matches in paraphrased elements.

In scientific research, the most time-consuming and challenging task can be the process of filtering out unnecessary papers on the one hand and identifying those that are needed on the other hand. This difficult yet critical task can be daunting. It discourages many researchers from participating in scientific research. If AI could replace this process, it will be easier to collect and analyze data from the selected papers. Recently, commercial literature search services using generative AI models have emerged. Representative examples include Covidence [29], Consensus [30], and Elicit [31]. The technical details of these commercial AI literature search services are unknown, but they are based on LLMs using GPT. Therefore, these search services are not only insufficient to verify hallucinations but also lack information in the search target databases. Even if there may be mistakes, the researcher should aim for completeness, and unverified methods should be avoided. Although this study did not use a commercial literature search service, it manually searched the target databases one by one. If the reliability and consistency of AI literature search services are verified, the use of these technologies will help medical research greatly

This study suggests that ChatGPT still has limitations in academic search, despite the recent assertion from Zhou et al [8] about its potential in searching for academic evidence. Moreover, although ChatGPT can search and identify guidance in open-access guidelines, its results are brief and fragmentary, often with just 1 or 2 sentences that lack relevant details about the guidelines.

Arguably, more concern should be placed on the potential use of ChatGPT in a CDSS than its role in education or writing draft papers. On the one hand, if AI such as ChatGPT is used within a patient-physician relationship, this is unlikely to affect liability since the advice is filtered through professionals’ judgment and inaccurate advice generated by AI is no different from erroneous or harmful information disseminated by a professional. However, ChatGPT lacks sufficient accuracy and speed to be used in this manner. On the other hand, ChatGPT could also be used to give direct-to-consumer advice, which is largely unregulated since asking AI directly for medical advice or emotional support acts outside the established patient-physician relationship [32]. Since there is a risk of patient knowing inaccurate information, the medical establishment should seek to educate patients and guardians about the risk of inaccurate information in this regard.

Academic interest in ChatGPT to date has mainly focused on potential benefits including research efficiency and education, drawbacks related to ethical issues such as plagiarism and the risk of bias, as well as security issues including data privacy. However, in terms of providing medical information and acting as a CDSS, the use of ChatGPT is currently less certain because its academic search capability is potentially inaccurate, which is a fundamental issue that must be addressed.

The limitation of this study is that it did not address various research topics, because only 1 research topic was searched when collecting target literature. In addition, due to the time difference between the start of the study and the review and evaluation period, the latest technology could not be fully applied because it could become an outdated technology in a field of study where technology advances rapidly, such as generative AI. For example, there have already been significant technological advances since new AI models such as ChatGPT Turbo (4.0) were released between the time we started this study and the current revised time point.

This paper thus suggests that the use of AI as a tool for generating real-time evidence for a CDSS is a dream that has not yet become a reality. The starting point of evidence generation is a systematic search and ChatGPT is unsuccessful even for this initial purpose. Furthermore, its potential use in providing advice directly to patients in a direct-to-consumer form is concerning, since ChatGPT could provide inaccurate medical information that is not evidence based and can result in harm. For the proper use of generative AI in medical care in the future, it is suggested that a feedback model that evaluates accuracy according to experts’ perspective, as done in this study, and then reflects it back into an LLM is necessary.

### Conclusion

This is the first study to compare AI and conventional human SR methods as a real-time literature collection tool for EBM. The results suggest that the use of ChatGPT as a tool for real-time evidence generation is not yet accurate and feasible. Therefore, researchers should be cautious about using such AI. The limitations of this study using the GPT model are that the search for research topics was not diverse and that it did not prevent the hallucinations of generative AI. However, this study will serve as a standard for future studies by providing an index to verify the reliability and consistency of generative AI from a user’s point of view. If the reliability and consistency of AI literature search services are verified, the use of these technologies will help medical research greatly.

## Supplementary material

10.2196/51187 Multimedia Appendix 1 Additional logical flow diagrams, characteristics of studies included in Lee et al [9], ChatGPT and Microsoft Bing transcripts, and grade classification for answers.

## Abbreviations

AI: artificial intelligence
CDSS: clinical decision support system
EBM: evidence-based medicine
GPT: generative pre-trained transformer
LLM: large language model
PD: Peyronie disease
RCT: randomized controlled trial
SR: systematic review
